# Reinforcement of Castor Oil-Based Polyurethane with Surface Modification of Attapulgite

**DOI:** 10.3390/polym10111236

**Published:** 2018-11-07

**Authors:** Chengshuang Wang, Lili Dai, Zhengrui Yang, Chengcheng Ge, Shuiping Li, Meng He, Liang Ding, Hongfeng Xie

**Affiliations:** 1 School of Materials Science and Engineering, Yancheng 224051, China; dll081218@163.com (L.D.); 15190638055@163.com (Z.Y.); gcc13626213109@163.com (C.G.); lishuiping2002@126.com (S.L.); hemeng315@163.com (M.H.); dl1984911@ycit.edu.cn (L.D.); 2 You Pei College, Yancheng Institute of Technology, Yancheng 224051, China; 3 Department of Chemical and Environmental Engineering, University of California-Riverside, Riverside, CA 92521, USA; 4 Key Laboratory of High Performance Polymer Materials and Technology (Nanjing University), Ministry of Education, School of Chemistry and Chemical Engineering, Nanjing University, Nanjing 210093, China

**Keywords:** polyurethane, castor oil, attapulgite, nanocomposites, reinforcement

## Abstract

Polyurethane/attapulgite (PU/ATT) nanocomposites derived from castor oil were prepared by incorporation of 8 wt % ATT, acid-treated ATT, and KH560-treated ATT. The effects of three ATTs (ATT, acid-ATT, and KH560-ATT) on the comprehensive properties of PU/ATT nanocomposites were systematically investigated. The results showed that the incorporation of 8 wt % of three ATTs could produce an obvious reinforcement on the castor oil-based PU and that the silane modification treatment, rather than the acid treatment, has the more effective reinforcement effect. SEM images revealed the uniform dispersion of ATT in the PU matrix. DMA confirmed that the storage modulus and glass transition temperature (*T*_g_) of PU/ATT nanocomposites were significantly increased after blending with different ATTs. For PU/KH560-ATT8 nanocomposites, the thermal stability of the PU was obviously enhanced by the addition of KH560-ATT. In particular, 8 wt % KH560-ATT loaded castor oil-based PU nanocomposites exhibit an obvious improvement in tensile strength (255%), Young’s modulus (200%), *T*_g_ (5.1 °C), the storage modulus at 25 °C (104%), and the initial decomposition temperature (7.7 °C). The prepared bio-based PU materials could be a potential candidate to replace petroleum-based PU products in practical applications.

## 1. Introduction

Recently, the use of renewable vegetable oil-based polyols to replace the commercial petroleum-based polyether or polyester polyols in the synthesis of bio-based polyurethane (PU) [1,2,3,4,5] has attracted great public attention as functional materials in many applications, such as thermoplastic [6,7], thermosets elastomers [8,9], adhesives [10,11,12], coatings [13,14,15], foams [16,17], medical fields [18,19] and nanocomposites [20,21]. The advantages of these bio-based materials are their biocompatibility, biodegradability, low cost, good thermal properties, and acceptable specific strength properties [22,23]. In comparison to other vegetable oils, castor oil has on average 2.7 hydroxyls per triglyceride and can therefore be directly used to synthesize PU materials [24]. However, the resulting pure castor oil-based PU is too soft to meet application requirements in some fields. This may be attributed to the intrinsic low OH value of castor oil and low cross-linking density of PU matrix [25,26].

Considering the problem of the low mechanical strength of PU materials derived from castor oil, the direct strategy is to incorporate functional nanofillers into the PU matrix. The functional nanofillers can be one-dimensional, such as fibers and nanotubes; two-dimensional, such as layered silicate minerals like clay; and three-dimensional, such as cubical and spherical nanoparticles. Thus, clays, such as montmorillonite [27,28,29] and attapulgite (ATT) [30,31], carbon nanotubes [32], reduced graphene oxide [33,34], cellulose nanowhiskers [35] and cellulose nanocrystals [36,37,38], a large number of nanoparticles of metals, and their oxides [39,40,41,42,43,44,45], are used as nanomaterials in the preparation of bio-based PU nanocomposites.

Among various clays, ATT, ideally (Si_8_O_20_Mg_5_(Al)(OH)_2_(OH_2_)_4_·4H_2_O), is a natural and low cost silicate mineral with a broad range of applications [46], and due to its ready availability, low cost and unique fibrous structure, has great potential application in the field of bio-based PU high performance nanocomposites. To ensure sufficiently uniform dispersion of the ATT in the bio-based PU matrix and to increase the interfacial interactions between the ATT and the PU matrix, a surface modification of the ATT is typically required. ATT is commonly modified through the reaction between an organic modifier and the hydroxyl groups of ATT [47,48,49]. Thus, the functionalized ATT, together with a silane coupling agent, is usually used in the preparation of high-performance PU nanocomposites [28,31,50]. The silane-treated ATT could strengthen the interfacial interactions and the efficient interfacial stress transfer between the ATT and PU matrix.

In our study, the ATT was successively modified by acid treatment and by KH560 surface modification. Then, PU nanocomposites based on castor oil were prepared by incorporation of 8 wt % different ATTs via in situ polymerization. The resulting ATT-reinforced PU nanocomposites were systematically investigated by their mechanical, thermal properties and micro-morphology.

## 2. Materials and Methods

### 2.1. Materials

KH560 (3-glycidoxypropyltrimethoxysilane) was purchased from the Jintan Fanshi Organosilicone Co., Ltd. (Jiangsu, China). The raw ATT was supplied by the Jiangsu Goldstone Attapulgite R&D Co., Ltd. (Jiangsu, China). Analytical-grade acetone, toluene, isopropyl alcohol, acetic acid, hydrochloric acid, and castor oil were all purchased from Shanghai Energy Chemical Co., Ltd. (Shanghai, China). The OH value of castor oil was 160 mg KOH g^−1^, which was determined according to the previous report [44]. Isophorone diisocyanate (IPDI) was obtained from Shanghai Aladdin Biochemical Technology Co., Ltd. (Shanghai, China).

### 2.2. Synthesis of Acid-ATT and KH560-ATT

The raw ATT (10 wt %) was dispersed in 1 mol/L hydrochloric acid solution by sonication for 90 min at 250 W. The ATT was activated to introduce more -OH groups on the surface. After exhaustively washing with water and acetone until pH neutral, the acid-ATT was dried at 120 °C for 12 h in vacuum to remove water. For KH560-ATT, the acid-ATT (6 g) was dispersed in 120 mL toluene by sonication for 30 min at 250 W. The 20 mL KH560 was added to the 80 mL isopropyl alcohol/water mixture (*v*/*v* = 9/1). The pH of the KH560 mixture solution was adjusted at around 34 by acetic acid. The ATT dispersion was then mixed with the KH560 hydrolysis solution, refluxing under electromagnetic stirring at 110 °C for 4 h. Finally, the residue was washed six times with toluene and acetone to remove the unreacted KH560. Once obtained, the KH560-ATT was dried at 120 °C for 12 h in vacuum.

### 2.3. Synthesis of Castor Oil-Based PU Nanocomposites

The neat PU was synthesized by the reaction of IPDI with castor oil according to our previously described method [44]. The PU/ATT nanocomposites were prepared as follows: the three ATTs (ATT, acid-ATT, and KH560-ATT) were respectively added in dry acetone and the obtained suspension was sonicated for 60 min. The castor oil was added and stirred vigorously at room temperature (RT) for 24 h. The acetone was completely removed at 80 °C. The IPDI was then added and stirred for 30 min while cooling to RT. The mixtures with 8 wt % loading of three ATTs were poured to a PTFE (Teflon) mold of 100 mm diameter and 4 mm depth and degassed under vacuum until all bubbles disappeared. The samples were first cured at 90 °C for 2 h and then 110 °C for 24 h. Finally, the samples were slowly cooled down to RT and demolded. The resulting PU nanocomposites with 8 wt % ATT, acid-ATT, and KH560-ATT were denoted as PU/ATT8, PU/acid-ATT8 and PU/KH560-ATT8, respectively.

### 2.4. Characterization

X-ray diffraction (XRD) patterns was recorded by a XRD-6000 (Shimadzu, Kyoto, Japan) with a crystal monochromated Cu *K*_α_ radiation (λ = 0.154 nm), the scanning rate was 6°·min^−1^ from 6° to 60°. Dynamic mechanical analysis (DMA) was measured by a DMA+450 (01dB-Metravib, Limonest, France) under tension mode at a fixed frequency (1 Hz) with a heating rate of 2 °C·min^−1^ from −70 to 50 °C for each sample. The mechanical performances of PU/ATT nanocomposites were performed at 23 °C using an 4466 universal testing machine (Instron, Norwood, MA, USA) at a strain rate of 50 mm·min^−1^ based on ASTM D638 standard. At least six specimens were tested for obtaining the average value of mechanical results. Thermogravimetric analysis (TGA) were performed under a N_2_ atmosphere with a TGA/DSC 1 STAR^e^ system (Mettler-Toledo, Greifensee, Switzerland) at a heating rate of 20 °C·min^−1^ in the temperature range 30–600 °C. Scanning electron microscopy (SEM) was observed by an S-4800 instrument (Hitachi, Tokyo, Japan), the acceleration voltage of the electron beam was 3 kV. The liquid nitrogen fractured surface of the samples was sputter coated with gold to provide enhanced conductivity before observation.

## 3. Results

### 3.1. X-ray Diffraction (XRD) Analysis

**Figure 1:**
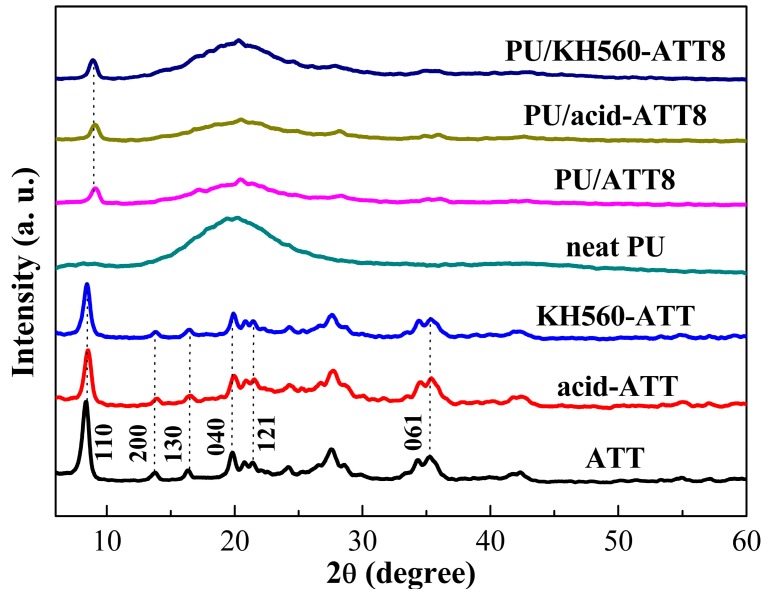
The XRD patterns of different ATTs, neat PU and PU nanocomposites containing 8 wt % ATT, acid-ATT and KH560-ATT.

XRD has been a useful tool for characterizing the structure of clay and its nanocomposites. The XRD patterns of different ATTs and PU/ATT nanocomposites were presented in Figure 1. From the diffractograms, the typical diffraction peaks at 2*θ* = 8.4°, 13.8°, 16.4°, 19.8°, 21.4° and 35.3° are attributed to the diffraction of the (110), (200), (130), (040), (121) and (061) planes of the ATT, respectively. The XRD patterns of the neat ATT clearly exhibited the six-plane characteristic peaks. The characteristic reflection of both acid-ATT and KH560-ATT do not change at all. The results indicated that the crystal structure of ATT did not alter or distort after acid-treated and silylated surface modification. As shown in Figure 1, the castor oil-based PU exhibited a strong broad diffraction peak at 2*θ* = 20.0°, which is caused by the short-range order in the arrangement of amorphous PU chain segments. For the PU/ATT nanocomposites, the new characteristic diffraction peak at 2*θ* = 9.0° is attributed to the characteristic diffraction peak of ATT in the PU matrix. The results demonstrated that the blended ATTs in the PU matrix could keep their intrinsic crystal structures.

### 3.2. Dynamic Mechanical Properties

**Figure 2:**
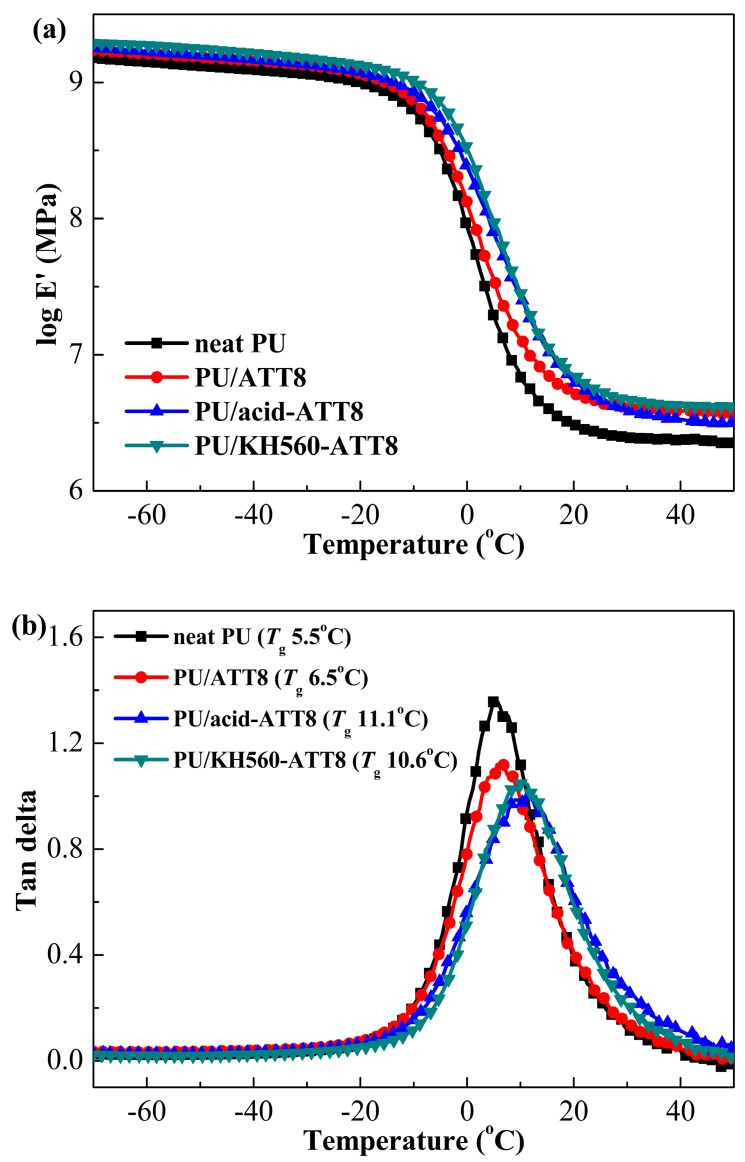
Dynamic mechanical spectra in terms of logarithmic *E′* (**a**) and tan *δ* (**b**) for neat PU and PU nanocomposites containing 8 wt % ATT, acid-ATT and KH560-ATT.

DMA can provide insight into the effect of surface modified ATTs on the thermo-mechanical performances of the obtained PU/ATT nanocomposites. Figure 2 showed the temperature dependence of the storage modulus (logarithmic *E′*) and the loss tangent (tan *δ*) for neat PU and PU nanocomposites containing 8 wt % ATT, acid-ATT and KH560-ATT. The dynamic mechanical spectra regarding the logarithmic *E′* were very similar for castor oil-based ATT-reinforced PU nanocomposites. A steep decrease of logarithmic *E′* is observed, corresponding to improved chain mobility in the PU matrix upon transition through glass transition temperature (*T*_g_). The logarithmic *E′* curves that demonstrated the logarithmic *E′* value of PU/ATT nanocomposites within the whole test temperature range were indeed larger than that of neat castor oil-based PU, which indicated the effective reinforcement of three ATTs in the PU matrix. The neat castor oil-based PU had an *E′* at 25 °C (*E′*_25_) of 2.64 MPa. The *E′*_25_ of PU/ATT8 and PU/acid-ATT8 was increased 70% and 77% compared with that of neat PU, respectively. The maximum improvement (increased 104%) in *E′*_25_ is observed for PU/KH560-ATT8 with 8 wt % KH560-ATT. Such significant improvement in *E′*_25_ is likely to be due to good compatibility and interphase interaction between KH560-ATT and the PU matrix.

The *T*_gs_ of neat PU and PU nanocomposites containing 8 wt % ATT, acid-ATT and KH560-ATT were presented in Figure 2b and Table 1. It is well known that *T*_g_ reveals the segmental mobility of polymer chains in the matrix. Generally, an enhanced *T*_g_ indicated that the segmental mobility of the polymer chains decreased [51]. It is noted that three ATTs in general increased the *T*_g_ of PU/ATT nanocomposites. Compared with the *T*_g_ of the neat PU (5.5 °C), the *T*_gs_ of PU/acid-ATT8 and PU/KH560-ATT8 were respectively improved by 5.6 and 5.1 °C. The improvement can be attributed to both acid-ATT and KH560-ATT decreased segmental mobility of the castor oil-based PU chains and the enhanced interfacial interaction between the ATTs and the PU matrix.

Usually, the thermo-mechanical properties of thermosets usually were strongly associated with crosslink density (*ν*_e_). Based on the kinetic theory of rubber elasticity [52], the *ν*_e_ of the cured crosslinking systems can be calculated from the rubbery *E′* using Equation (1):

*E′* = 3 *ν*_e_*RT* (1)

where *E′* represents the storage modulus of cured crosslinking systems in the rubbery plateau region, *T* is the absolute temperature, and *R* is the gas constant. In this study, the rubber modulus at *T*_g_ + 40 °C was selected for the calculation of *ν*_e_. The data for *E′* at 50 °C (*E′*_50_) and the calculated *ν*_e_ of PU/ATT nanocomposites are summarized in Table 1. The value of *ν*_e_ for castor oil-based PU was 269.2 mol/m^3^. It is noted that the *ν*_e_ of PU/KH560-ATT8 was increased to 513.6 mol/m^3^ (increased by 91%). Furthermore, the *ν*_e_ of PU/ATT8 and PU/acid-ATT8 was respectively increased 69% and 40%, compared with that of neat PU.

### 3.3. Mechanical Properties

**Figure 3:**
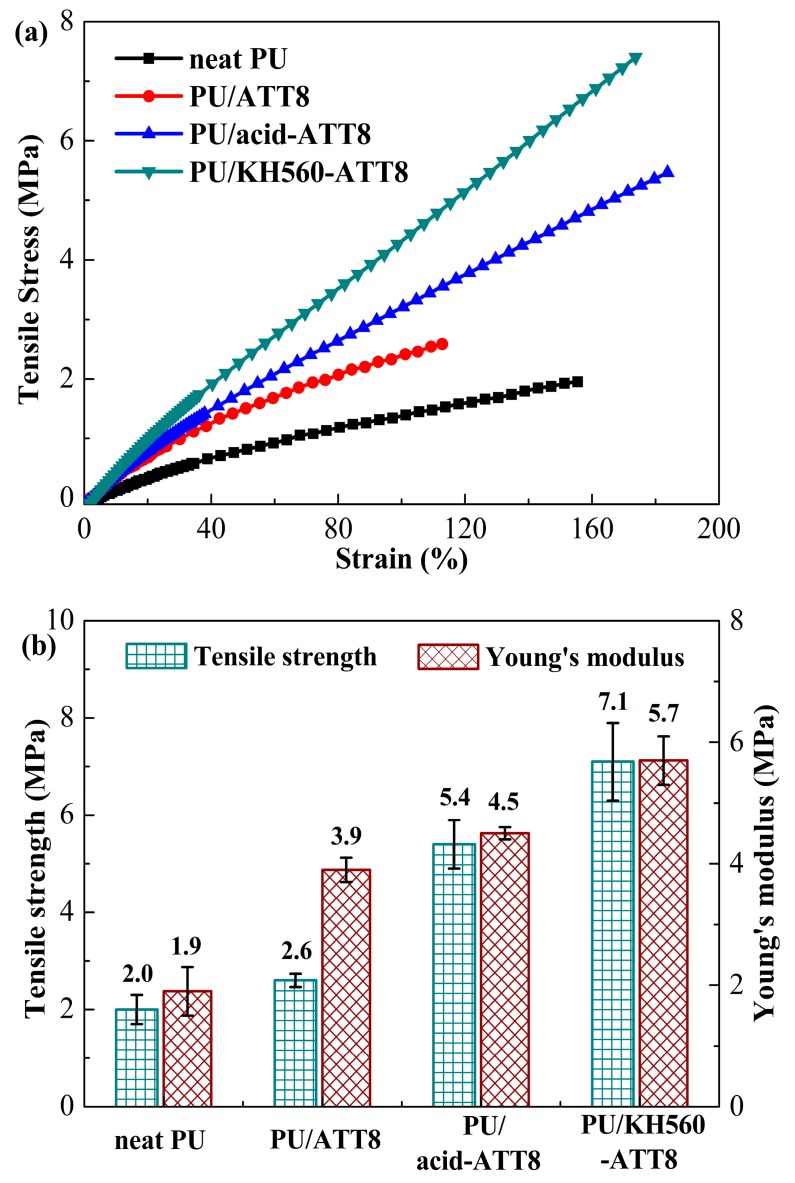
(**a**) Stress-strain curves of neat PU and PU nanocomposites containing 8 wt % ATT, acid-ATT and KH560-ATT; (**b**) effect of different ATTs on the tensile strength and Young’s modulus of PU nanocomposites containing 8 wt % ATT, acid-ATT and KH560-ATT.

For superior PU nanocomposites, the mechanical performances of PU nanocomposites are significantly influenced by the nanofiller dispersion and interfacial interaction between nanofiller with the PU matrix. The ATT surface modification via acid treatment and KH560 modification should be an effective strategy to improve ATTs’ dispersion and load transfer efficiency. The stress–strain curves and tensile performances of neat PU and PU nanocomposites containing 8 wt % ATT, acid-ATT and KH560-ATT were presented in Figure 3 and the detailed data were listed in Table 2. From the curves in Figure 3a, neat PU and three PU nanocomposites showed similar linear elastic deformation behavior. The neat PU had a tensile strength of 2.0 MPa and a Young’s modulus of 1.9 MPa. The tensile strength and Young’s modulus of PU/ATT nanocomposites were dramatically improved with incorporation of different ATTs into the PU matrix. The maximum improvement in the tensile strength (255%) and Young’s modulus (200%) was observed for PU/KH560-ATT8, which is likely to be due to better homogeneous dispersion and good compatibility between KH560-ATT and the PU matrix. The PU/ATT8 and PU/acid-ATT8 had a 30% and 170% increment in the tensile strength, and had a 105% and 137% increment in Young’s modulus, respectively. The increase of tensile strength indicated that three ATTs have an obvious reinforcement effect on the PU matrix, which were consistent with the DMA results. The reinforcement effects of different ATTs are in the order of KH560-ATT > acid-ATT > ATT and thus the KH560-ATT has the best reinforcement effect among three ATTs. It is noted that the elongation at break of PU/acid-ATT8 and PU/KH560-ATT8 were higher than that of neat PU. The elongation at break slightly improved because the decrease of ductility was not significant after incorporation of homogeneous dispersed ATTs, which contributed to a tougher PU matrix.

### 3.4. Thermal Stability

**Figure 4:**
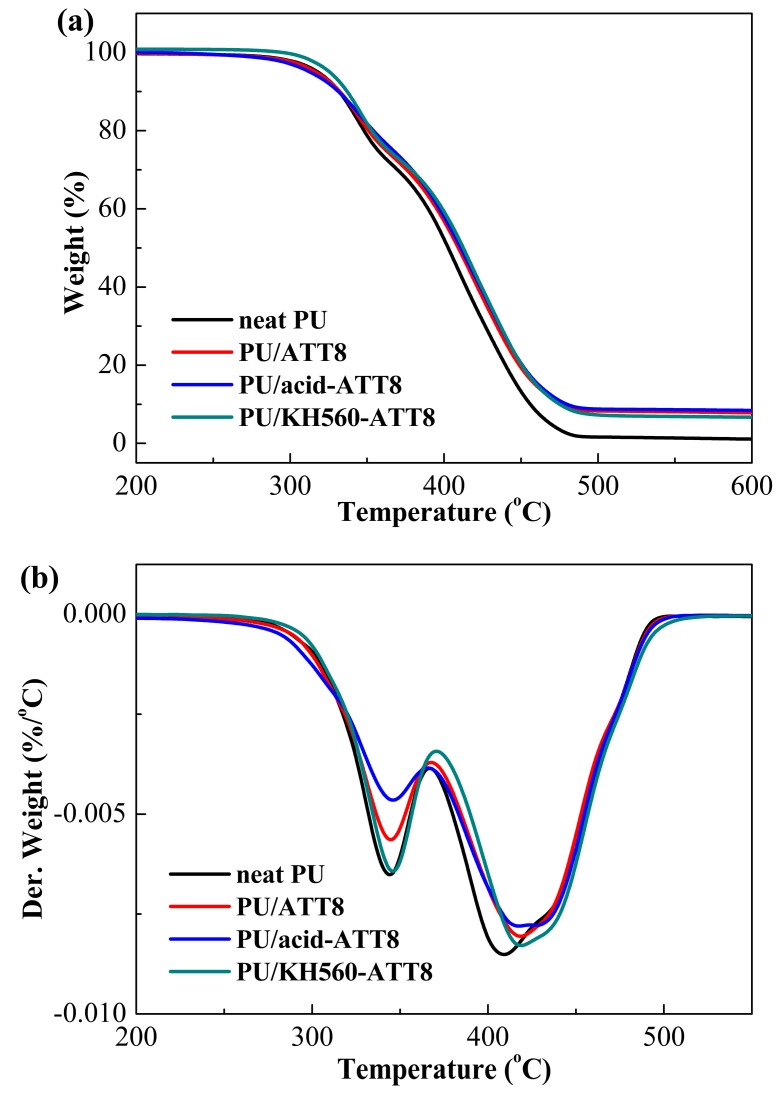
TGA (**a**) and DTG (**b**) curves of neat PU and PU nanocomposites containing 8 wt % ATT, acid-ATT and KH560-ATT.

**Figure 5:**
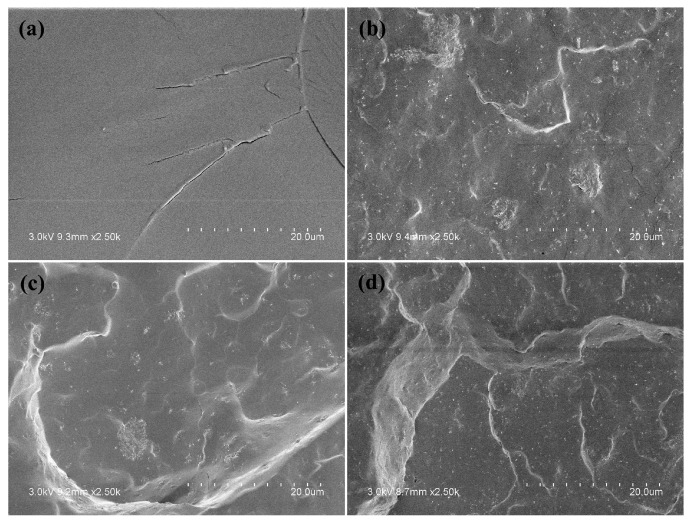
SEM images of neat PU and PU nanocomposites: neat PU (**a**), PU/ATT8 (**b**), PU/acid-ATT8 (**c**) and PU/KH560-ATT (**d**).

The thermal stabilities of neat PU and PU nanocomposites containing 8 wt % ATT, acid-ATT and KH560-ATT were investigated with TGA in nitrogen. Figure 4 displays the TGA thermograms versus temperature of neat PU and PU/ATT nanocomposites. The TGA and DTG curves were analyzed and the data were listed in Table 3. The addition of 8 wt % KH560-ATT into the PU matrix remarkably improved the IDT (5% mass loss temperature), *T*_10%_ and *T*_50%_ of PU. Especially, the IDT of PU/KH560-ATT8 was delayed by 7.7 °C compared with that of neat PU. The TGA results indicated that the incorporation of KH560 surface modified ATT into the PU matrix significantly improved its thermal stability. The increase in thermal stability was due to the barrier effect of well dispersed KH560-ATT (Figure 5), which delays the release of volatile degradation compounds from the PU matrix.

As can be seen in Figure 4b, the decomposition processes of PU/ATT nanocomposites were similar with that of the PU. The decomposition of neat PU and PU/ATT nanocomposites showed the typical decomposition steps [53,54]: the first loss step (230–368 °C) corresponds to degradation of urethane and urea groups in hard segment, while the second loss step (368–520 °C) can be due to the decomposition of the soft segment from castor oil. After the incorporation of different ATTs into the castor oil-based PU matrix, both of the decomposition steps of the PU/ATT nanocomposites were slightly subdued compared to that of neat PU. Besides, the *T*_max2_ (maximum rate of degradation temperature of second stage) and the char at high temperatures of PU/ATT nanocomposites were higher than that of the neat PU. The results indicated that different ATTs were well dispersed in the PU matrix and resulted in the shift of the *T*_max2_ to higher temperature. As shown in Table 3, the char at 600 °C (*w*_char_) of neat castor oil-based PU is 1.1%. The *w*_char_ of PU/ATT nanocomposites was larger than that of the neat PU, ranging from 6.7% to 8.4% as the 8 wt % different ATTs in the PU matrix. Usually, the thermal stability of polymer and its nanocomposites can be evaluated by the value of IDT and *w*_char_. Thus, the introduction of KH560-ATT into the PU matrix could obviously improve the thermal stability of castor oil-based PU nanocomposites, which is attributed to homogeneous KH560-ATT dispersion and strong interfacial interaction between KH560-ATT and the PU matrix.

### 3.5. Morphology

The morphologies and dispersion of the ATTs in the PU matrix were examined with SEM. The fractured micrographs are shown in Figure 5. The increase in roughness after addition of different ATTs into the PU matrix is the result of the deflection of the crack path induced by the presence of the rigid ATT in the path of the growing crack. The bright points were the broken ATTs, illustrating that most of the ATTs had broken apart from the fractured surface. Despite the existence of some aggregations in the PU/ATT8 and PU/acid-ATT8, the KH560-ATT dispersion was uniform throughout the PU matrix. The rougher surface and KH560-ATT uniform dispersion in Figure 5d indicates that KH560 surface modification could improve compatibility and interfacial interaction between ATT with the PU matrix. Hence, among the three ATTs, KH560-ATT had the most reinforcement effect on the PU matrix, which demonstrated the mechanical performance results of neat PU and PU/ATT nanocomposites.

## 4. Conclusions

PU nanocomposites based on castor oil were prepared by incorporating neat ATT, acid-ATT, and KH560-ATT into the PU matrix. The results showed that the incorporation of 8 wt % of the three ATTs could result in a significant reinforcement on the castor oil-based PU. SEM revealed that KH560 surface modified ATT enhanced the dispersion of KH560-ATT in the PU matrix. DMA confirmed that the 8 wt % KH560-ATT loaded castor oil-based PU nanocomposite exhibits the obvious improvements in the storage modulus at 25 °C (104%) and *T*_g_ (5.1 °C). Remarkably, at 8 wt % KH560-ATT loading, the 255% increase in tensile strength, 200% increment in Young’s modulus were obtained. Furthermore, the thermal stability of PU/KH560-ATT8 was significantly enhanced and the initial decomposition temperature increased by 7.7 °C. This work may provide an effective and potential approach to develop bio-based PU materials for practical applications.

## Figures and Tables

**Table 1:** Dynamic mechanical properties of neat PU and PU nanocomposites containing 8 wt % ATT, acid-ATT and KH560-ATT.

Sample	*E′*_25_^a^ (MPa)	*E′*_50_^b^ (MPa)	*T*_g_^c^ (°C)	*ν*_e_^d^ (mol/m^3^)
neat PU	2.64	2.17	5.5	269.2
PU/ATT8	4.50	3.67	6.5	455.3
PU/acid-ATT8	4.68	3.05	11.1	378.4
PU/KH560-ATT8	5.38	4.14	10.6	513.6

^a^ Storage modulus at 25 °C; ^b^ Storage modulus at 50 °C; ^c^ Glass transition temperature; ^d^ Crosslink density.

**Table 2:** Mechanical properties of neat PU and PU nanocomposites containing 8 wt % ATT, acid-ATT and KH560-ATT.

Sample	Tensile Strength at Break (MPa)	Elongation at Break (%)	Young’s Modulus (MPa)
neat PU	2.0 ± 0.3	164.4 ± 17.5	1.9 ± 0.4
PU/ATT8	2.6 ± 0.1	110.7 ± 5.5	3.9 ± 0.2
PU/acid-ATT8	5.4 ± 0.5	179.1 ± 17.0	4.5 ± 0.1
PU/KH560-ATT8	7.1 ± 0.8	167.9 ± 22.3	5.7 ± 0.4

**Table 3:** TGA and DTG results for neat PU and PU nanocomposites containing 8 wt % ATT, acid-ATT and KH560-ATT.

Sample	IDT ^a^ (°C)	*T*_10%_^b^ (°C)	*T*_50%_^c^ (°C)	*T*_1max_^d^ (°C)	*T*_2max_^e^ (°C)	*w*_char_^f^ (%)
neat PU	318.0	331.9	402.7	344.1	409.0	1.1
PU/ATT8	316.7	332.3	409.3	344.8	418.6	7.8
PU/acid-ATT8	312.9	331.4	410.9	346.1	418.2	8.4
PU/KH560-ATT8	325.7	337.1	412.5	345.8	418.9	6.7

^a^ Initial decomposition temperature, 5% mass loss temperature; ^b^ 10% mass loss temperature; ^c^ 50% mass loss temperature; ^d^ Maximum rate of degradation temperature of first stage; ^e^ Maximum rate of degradation temperature of second stage; ^f^ Char at 600 °C.
